# Maternal biological ageing and telomere attrition across parity, pregnancy, and the postpartum period

**DOI:** 10.1007/s10522-026-10487-0

**Published:** 2026-08-10

**Authors:** Laura Collopy, Yana Kolenichenko

**Affiliations:** Faculty of Medicine, National Heart and Lung Institute, Imperial College London, Guy Scadding Building, Cale Street, London, SW3 6PT UK

**Keywords:** Biological ageing, Telomere length, Epigenetic ageing, Pregnancy, Parity, Postpartum period

## Abstract

Telomere length (TL) is a well-established biomarker of biological ageing, sensitive to cumulative physiological and psychosocial stress. This review synthesises current evidence on how pregnancy, postpartum stressors, and reproductive history shape maternal biological ageing, integrating findings from telomere biology and emerging epigenetic ageing measures. Pregnancy represents a period of substantial metabolic, hormonal, and immunological demand and is increasingly conceptualised as a transient state of accelerated biological ageing. While telomere shortening is not consistently detectable during gestation, epigenetic clocks indicate a temporary increase in biological age, which is only partially reversible postpartum. Across the life course, higher parity is associated with shorter TL, with evidence suggesting a cumulative effect that becomes most apparent in later life and around the menopausal transition. However, this relationship is heterogeneous and modified by factors including age at last birth, breastfeeding, and socioeconomic context. Postpartum represents a critical and underexplored window in which sleep deprivation, psychological stress, and social factors converge to influence telomere dynamics. In particular, poor sleep quality and postpartum depression (PPD) are consistently linked to accelerated telomere attrition and epigenetic ageing, with emerging evidence of a bidirectional relationships whereby shorter TL may also predispose to PPD. Overall, evidence supports a model in which reproductive events impose are associated with measurable transient and cumulative costs to cellular ageing biomarkers. These findings highlight the importance of incorporating postpartum health, particularly sleep and mental health support, into life-course models of ageing and underscore the need for longitudinal, mechanistic, and intervention-focused research in maternal populations.

## Introduction

Telomeres are repetitive DNA sequences, composed of tandem repeats of the hexanucleotide sequence (TTAGGG)n, that cap and protect the ends of linear chromosomes. Together with associated shelterin proteins, they play a critical role in maintaining genomic stability by preventing chromosomal end-to-end fusion and degradation. Due to the end-replication problem, inherent in DNA polymerase activity, telomeres progressively shorten with each cell division, ultimately leading to cellular senescence or apoptosis when critically short lengths are reached. As such, telomere length (TL) has become an established biomarker of biological ageing and cumulative cellular stress (Blackburn et al. 2015).

The rate of telomere shortening is not solely determined by replicative history but is also influenced by external and internal stressors. Oxidative damage is a major driver of telomere shortening, operating through a mechanism in which 8-oxoguanine lesions in telomeric sequences impair replication fork progression (von Zgliniki et al. 2002). Similarly, chronic inflammation contributes to increased cellular turnover and the production of reactive oxygen species (ROS), further exacerbating shortening (Zhang et al. 2016). Hormonal factors, including glucocorticoids, have also been implicated in modulating telomerase activity, with elevated cortisol in response to psychosocial stress demonstrably associated with shorter leukocyte telomere length (LTL, Epel et al. 2004). These influences are particularly relevant in the context of pregnancy and the postpartum period, which together constitute one of the most physiologically demanding phases of the human lifespan (Grzeszczak et al. 2023). The postpartum period is marked by substantial hormonal shifts, immune modulation, and behavioural changes, most notably profound disruption of sleep patterns. Recent work has begun to frame pregnancy itself as a transient model of accelerated ageing (Pham et al. 2024; Ryan et al. 2024).

A 2023 meta-analysis pooling data from 414 study samples including 743,019 individuals, confirmed a robust inverse association between telomere length and chronological age, with an average attrition rate of approximately 38 base pairs per year across adulthood. The relationship is non-linear, with the steepest attrition occurring during early childhood, followed by relative stabilisation and then more gradual shortening (Ye et al. 2023). This non-linearity has important implications for understanding how specific life stages, including pregnancy, the postpartum period and perimenopause, interact with baseline telomere biology and population-level reference ranges. Shortened telomeres have been associated with a wide range of age-related diseases, including cardiovascular disease, type diabetes, neurodegenerative disorders, and certain cancers (Gruber et al. 2021). Furthermore, telomere length has been linked to all-cause mortality, reinforcing its utility as a biomarker of biological ageing (Cawthon et al. 2003).

This review seeks to evaluate several long-standing open problems in general biogerontology. Rattan (2024) and Talay et al. 2026 have identified key priorities in biogerontology, including biomarkers of intrinsic biological age, sex-specific variation in ageing mechanisms, and measurement of the homeodynamic space (Rattan 2024; Talay et al. 2026). Human reproduction offers an excellent model in which to interrogate these gaps. As such, this review considers the cumulative impact of reproductive history, age at last childbirth, mode of delivery, and breastfeeding history, on long-term telomere biology (as a biomarker of ageing), integrating findings from molecular, epidemiological, and clinical studies, while identifying key gaps for future investigation.

### Measures of biological ageing

Telomere length is one of the most widely studied biomarkers of biological ageing, but it is not the only one. Over the past decade, a family of complementary tools known as epigenetic clocks has emerged from the observation that DNA methylation changes in highly predictable ways as humans age (Li et al. 2022). These methylation patterns can be measured from a standard blood sample and used to calculate a biological age estimate that may diverge substantially from chronological age. The degree of divergence, termed *epigenetic age acceleration*, is increasingly recognised as a powerful predictor of morbidity, mortality, and physiological decline. Three clocks are particularly prominent in the current literature.

PhenoAge represents a *second-generation* epigenetic clock, because it was designed to capture biological ageing as it relates to health outcomes and mortality risk, rather than simply predicting chronological age (Levine et al. 2018). It uses a composite of nine routine blood biomarkers to produce a *phenotypic age* score, which is used as the training target for a machine learning model applied to DNA methylation data from an Italian ageing cohort (the InCHIANTI study). Elastic net regression identified 513 CpG sites whose methylation levels collectively predicted phenotypic age. When an individual’s phenotypic age exceeds their chronological age, this epigenetic age acceleration indicates poorer biological health (Levine et al. 2018).

GrimAge (Lu et al. 2019a, b) takes a different approach and considers *time-to-death* data, constructed as a composite of seven DNA methylation-based surrogates for plasma proteins, together with a methylation-based estimate of smoking pack-years. Each of the proteins has established associations with ageing, inflammation, cardiovascular disease, or metabolic dysfunction. DunedinPACE (Belsky et al. 2022) is a conceptually distinct *third generation* epigenetic clock. Rather than estimating biological age as a static quantity (i.e. a number of years), it estimates the current rate at which a person is ageing, derived from 20 years of longitudinal multi-organ data. Between ages 26 and 45, participants born in the same year were measured repeatedly on 19 biomarkers spanning cardiovascular, metabolic, renal, hepatic, immune, dental, and pulmonary functioning. Linear mixed-effects models were used to estimate each participant’s personal rate of change across these systems, yielding a composite *pace of ageing* phenotype.

Used in combination, these three clocks capture distinct biological dimensions: current physiological age (PhenoAge), mortality risk through specific protein pathways (GrimAge), and dynamic pace of multi-organ decline (DunedinPACE). Together with telomere length, they represent the emerging gold standard for multi-biomarker assessment of biological ageing in maternal health research. TL itself can also be estimated epigenetically from the methylation levels of 140 CpG sites (DNAmTL, Lu et al. 2019a, b). Length estimates are provided in kilobases, and results correlate somewhat with directly measured LTL using traditional methods (qPCR, FISH, Southern blotting). DNAmTL, however, is particularly sensitive to lifestyle exposures such as smoking, diet, and physical activity, making it a useful tool in assessing the impact of childbearing in biological ageing.

It is important to emphasise that TL and the epigenetic clocks correlate with, rather than act as established causal drivers, of ageing. Furthermore, these biomarkers frequently diverge from one another: TL and DNA methylation age are often only weakly correlated indicating that they index partially distinct, imperfectly overlapping components of a still poorly defined ageing process, rather than a single unitary biological age (Shirazi et al. 2020). Throughout this review, associations between reproductive events and TL or epigenetic age acceleration are therefore best interpreted as evidence that reproduction leaves a measurable molecular signature consistent with accelerated cellular ageing, rather than as proof that telomere attrition is *the* (or even *a*) causal driver of maternal ageing more broadly.

### Reproductive history, and telomere length

A fundamental question in the field is whether bearing children is, in itself, associated with shorter telomeres, and whether women who have never had children retain longer telomeres over the long term. In a cross-sectional study using data from the National Health and Nutrition Examination Survey (NHANES), Pollack et al. (2018) examined LTL in 1,954 US women of reproductive age (20–44 years). After adjustment for age, race/ethnicity, BMI, socioeconomic status, education, and smoking, women with a history of live birth had LTL that was 4.2% shorter (95% CI 0.9–7.3%) than nulliparous women, an equivalent of approximately 116 fewer base pairs on average. Strikingly, this magnitude exceeded the telomere shortening attributable to obesity or smoking, suggesting that parity represents an independent and substantial contributor to accelerated cellular ageing.

This finding is consistent with the evolutionary framework of the disposable *soma theory*, which predicts that reproductive effort diverts energetic resources from somatic maintenance, thereby accelerating ageing (Kirkwood and Rose 1991). Under this model, the act of gestation and lactation may constitute a direct somatic cost to maternal cellular maintenance. Notably, the impact on biological ageing appears to be specific to mothers; fathers do not show an equivalent effect, supporting that it is the physical demands of gestation, childbirth and breastfeeding, not shared socioeconomic factors, that drive the results (Ryan et al. 2024).

Kresovich et al. (2018) demonstrated in the Sister Study (n = 1048 postmenopausal women), that increased parity was associated with shorter relative TL, with the association most pronounced for women with four or more births compared to those with zero or one birth. The same study also found that women who experienced a longer reproductive period (from menarche to final menstrual period) had shorter TL, an apparent paradox given the putative oestrogenic protection of telomerase, suggesting that the energetic and oxidative costs of repeated gestations and lactation may outweigh oestrogenic benefits when summed across the reproductive lifespan.

Ryan et al. (2018), studying young Filipino women (n = 821, aged 20–22) in the Cebu Longitudinal Health and Nutrition Survey, found that both TL and DNA methylation age acceleration were associated with gravidity (the total number of pregnancies): TL decreased (p = 0.031) and epigenetic age accelerated (p = 0.007) with increasing number of pregnancies. Crucially, neither measure at baseline predicted subsequent pregnancies over four years, supporting a causal direction from reproduction to cellular ageing rather than reverse causation.

The 2023 systematic review and meta-analysis by Houminer–Klepar,et al., pooled data from 14 studies and 2564 participants. They found a negative but non-significant association between parity and TL. Studies demonstrating a negative correlation tended to employ more rigorous methodological practices, and many null findings came from younger age groups, suggesting that the cumulative burden of childbirth on TL may manifest primarily in later life, particularly as women approach the perimenopausal transition. This age-moderation effect is an important observation: the physiological costs of repeated pregnancies may only become biologically legible in TL once the cumulative antioxidant depletion, immune activation, and energetic deficits accrued across multiple gestations and lactation periods exceed the buffering capacity of cellular repair mechanisms.

Ryan et al. (2024) further extended this evidence by demonstrating, in the Cebu cohort, that gravidity is associated not only with shorter TL but with accelerated epigenetic ageing across multiple clock systems, including GrimAge and DunedinPACE-equivalent measures. The study used DNAmTL to estimate TL, finding that women who had ever been pregnant showed significantly shorter telomere length estimates compared to never-pregnant women, with each additional pregnancy associated with a shortening of approximately 0.011 kilobases. This indicates that the cellular cost of higher parity can be detectable by the mid-twenties using sensitive multi-system ageing biomarkers. Notably, in the longitudinal analysis, the telomere measure did not show a statistically significant association with changes in pregnancy number, whereas the epigenetic clocks did, suggesting differential sensitivity across measurement modalities. These findings are consistent with the view that each additional pregnancy adds a quantifiable biological cost, even if the phenotypic expression of that cost in terms of disease risk may not emerge for decades.

The timing of childbirth, not merely its occurrence, appears to modulate TL. Fagan et al. (2017), analysing data from the Long Life Family Study, found that women with a later age at birth of their last child, specifically those who gave birth between ages 34–37 or after age 38, had significantly increased odds of being in the longest tertile of telomere length, compared with women who had their last child by age 29. This association strengthened as maternal age at last birth increased, suggesting a possible genetic underpinning: women who retain fertility into their late thirties may carry genetic variants associated with both a longer reproductive lifespan and more robust telomere maintenance.

Latour et al. (2020) also found that later maternal age at last birth was positively associated with longer LTL, with women giving birth at age 40 or older having notably longer telomeres than those whose last birth was before age 25 (n = 1232). The authors estimate this difference is equivalent to approximately nine years of biological ageing. There was suggestive evidence that the association may be strongest in women with only one or two live births, and in women who had ever used oral contraceptives. Given the cross-sectional design, two competing interpretations are possible: either later childbearing preserves telomere length, or, more likely given the existing literature, women with inherently longer telomeres are biologically better equipped to sustain pregnancy at a later age, consistent with the telomeric theory of reproductive senescence (Keefe et al. 2006). These findings sit in interesting contrast to studies such as Ryan et al. (2024), which found that pregnancy accelerates biological ageing, and suggest that the timing of reproduction may matter as much as the fact of it.

### Pregnancy as a model of accelerated ageing

The physiological demands of pregnancy create a sustained state of oxidative stress and immune activation that may directly affect maternal telomere integrity and rate of biological ageing. Pregnancy is characterised by increased metabolic demand, mitochondrial expansion, and elevated ROS production as a consequence of placentation and foetal growth (Fisher et al 2020). This is accompanied by a carefully modulated immune environment: it is tolerogenic in early pregnancy to prevent immunological rejection of the semi-allogeneic foetus, but progressively pro-inflammatory as gestation advances and labour approaches (Wang et al. 2024). A 2025 cross-sectional study of 88 women in their second and third trimesters found that oxidative stress and IL-6 were both independently associated with maternal TL (Etzel et al. 2025). Paradoxically, both oxidative stress and inflammation were positively associated with TL in this pregnant sample, a direction opposite to that observed in non-pregnant populations. The authors propose that this may reflect the unique physiological context of pregnancy, in which certain oxidative and inflammatory mediators serve adaptive roles in placental development and immune tolerance rather than purely damaging functions. This finding highlights the importance of not simply extrapolating from non-pregnant telomere-stress associations to the pregnant state and underlines the need for pregnancy-specific mechanistic frameworks (Etzel et al. 2025).

Using epigenetic clocks and telomere length data, Ryan et al. (2024) provided population-level evidence that gravidity is associated with accelerated epigenetic ageing, suggesting that reproductive history leaves a lasting molecular imprint. Pham et al. (2024) replicated and extended these findings in a US pregnancy cohort, showing that maternal biological age increased across gestation but was significantly reduced by three months postpartum. Critically, pre-pregnancy body mass index and breastfeeding emerged as important modifiers: higher BMI was associated with greater biological age increase, while exclusive breastfeeding was associated with a partial reversal of the pregnancy-associated epigenetic ageing signal postpartum (Pham et al. 2024). These findings suggest that the biological costs of pregnancy are not fixed but are modifiable through health behaviours, an insight with substantial clinical implications.

Earlier work in both animal models and humans demonstrated that biological age can be transiently accelerated by severe physiological stressors and subsequently partially restored upon recovery (Poganik et al. 2023). Pregnancy in this model functions as one such stressor, imposing a transient but measurable cost to somatic maintenance that is only partially reversed in the postpartum period. Rather than viewing pregnancy as simply accelerating ageing, it may be more accurate to describe it as causing a transient biological age increase that the body attempts to reverse postpartum, with the degree of recovery potentially shaping long-term ageing trajectories. Incomplete recovery from repeated pregnancies could therefore contribute to cumulative biological ageing over the life course.

The role of pregnancy complications as a source of additional telomere stress has been examined by Panelli et al. (2024) in a nested case–control study across two US academic institutions. Women with pre-eclampsia or spontaneous preterm birth did not show significantly different postpartum LTL compared with controls in this pilot study, though both conditions are associated with elevated oxidative stress and inflammation. The authors note that the sample was underpowered and that future adequately powered longitudinal studies are needed to determine whether pregnancy complications impose a lasting telomere cost beyond that attributable to uncomplicated pregnancy.

From a mechanistic standpoint, telomere shortening in *foetal* membranes at term parturition has been directly demonstrated by Phillippe et al. (2022), who proposed that ROS-driven telomere dysfunction in the foetal membranes acts as a molecular clock for the timing of spontaneous labour; an observation that positions telomere biology at the interface of reproductive physiology and timing of birth. While this is a feature of placental-foetal rather than maternal somatic biology, it illustrates the pervasive role of telomere-mediated cellular senescence in reproductive outcomes, and suggests that the reproductive tract as a whole may experience accelerated telomere dynamics during late pregnancy that has not yet been fully characterised in accessible maternal tissues.

When considering biological ageing, an important practical consideration concerns the choice of epigenetic clock for perinatal research. Ross et al. (2024) found that second-trimester GrimAge acceleration predicted shorter gestational age at birth and lower birthweight, suggesting that maternal epigenetic ageing has clinically meaningful foetal consequences. A growing body of evidence supports GrimAge and DunedinPACE as the most clinically informative clocks in maternal populations: GrimAge demonstrates the strongest concordance between blood and uterine myometrial tissue (Erickson et al. 2022), while DunedinPACE provides an estimate of the current rate of ageing, rather than a cumulative age estimate, making it particularly suited for capturing dynamic, period-specific ageing processes such as the postpartum year. Knight et al. (2022) found that GrimAge acceleration in granulosa cells was negatively associated with anti-Müllerian LTL.

Overall, pregnancy appears to induce a transient increase in biological ageing, reflected in both epigenetic markers and telomere dynamics, within a uniquely regulated oxidative and immunological environment. These changes may be partly reversible postpartum and are influenced by maternal health and behaviours, suggesting they are not biologically fixed. However, the extent to which repeated pregnancies or complications lead to lasting telomere attrition and cumulative ageing remains unclear, highlighting the need for longitudinal, pregnancy-specific models.

### Post-partum stressors and telomere biology

While pregnancy itself exerts significant effects on biological ageing, the physiological and psychosocial demands of the postpartum period also represent a critical yet often overlooked phase influencing maternal biological age. The postpartum period provides a naturalistic model for studying the effects of chronic sleep disruption on biological systems. New mothers frequently experience fragmented sleep, reduced total sleep duration, and irregular sleep–wake cycles due to infant feeding and caregiving demands (Bilodeau et al. 2025). Actigraphic studies document a mean reduction in total sleep time of 40–60 min per night during the early postpartum months, with marked fragmentation that may be more physiologically disruptive than equivalent reductions in healthy non-caregiving adults (Gay et al. 2004). These disruptions are most pronounced in the first six months postpartum but may persist well into the second year, particularly when infants experience sleep problems or when mothers are breastfeeding on demand (Bilodeau et al. 2025).

A substantial body of research has explored the relationship between sleep and telomere biology, though findings remain somewhat inconsistent. Observational studies have frequently reported associations between shorter sleep duration and reduced LTL. Prather et al. (2011) found, in healthy midlife women aged 49–66, that poorer sleep quality was associated with shorter LTL independent of age, BMI, race, and income. A 2024 meta-analysis incorporating 29 studies identified three specific sleep parameters significantly associated with telomere attrition: global Pittsburgh Sleep Quality Index scores, sleep-related daytime impairments, and wake-after-sleep-onset time, indicating that it is sleep continuity and quality, not merely duration, that underlie the cellular ageing relationship (Sabot et al. 2024).

Mechanistically, sleep deprivation activates multiple pathways that converge on telomere biology. It is associated with elevated levels of ROS and upregulation of the DNA damage response (DDR) and senescent-associated secretory phenotype (SASP) in older adults (Carroll et al. 2016). Sleep loss also promotes systemic inflammation through elevated IL-6 and tumour necrosis factor-alpha, and disrupts hormonal homeostasis, including cortisol and melatonin, both of which regulate telomerase activity (Broderick et al. 2025; Thomas et al. 2021). Longitudinal data demonstrate that poor sleep quality predicts greater telomere attrition over time in a population-based cohort, providing temporal support for a sleep-to-telomere causal pathway rather than merely a correlational one (Tempaku et al. 2025).

During the Healthy Babies Before Birth (HB3) study (n = 33), a longitudinal study of maternal mental health, women obtaining fewer than seven hours of sleep per night at six months postpartum showed significantly accelerated intrinsic epigenetic age, phenotypic epigenetic age and shorter DNAmTL at twelve months postpartum, with effect sizes corresponding to multiple years of additional biological ageing (Carroll et al. 2021). These findings suggest that the trajectory of biological recovery from pregnancy is critically dependent on the quality of postpartum sleep. More than half of participants were obtaining inadequate sleep at both six and twelve months postpartum. Importantly, sleep duration at twelve months was not itself significantly predictive, suggesting that the critical window is the accumulation of sleep debt during the most acutely deprived early postpartum months.

Complementary evidence from Mendelian randomisation analyses suggests a potential causal relationship: genetic variants that predispose to shorter sleep duration appear to contribute to telomere shortening, partially addressing concerns about reverse causation (Hu et al. 2023). Circadian rhythm disruption, as experienced by shift workers, has been independently associated with shorter telomeres, mediated through dysregulation of clock genes, impaired DNA repair mechanisms, and increased oxidative stress (Ferrari et al. 2025).

Panelli et al. (2022) contributed important mechanistic context from a pilot longitudinal study of nulliparous women with uncomplicated term pregnancies. Maternal LTL did not significantly change across gestation itself, but postpartum LTL was significantly shorter following caesarean delivery compared with vaginal birth (T/S ratio: 0.94 vs 1.12, p = 0.01). Furthermore, poor sleep quality was the primary stressor associated with shorter LTL both at baseline and on average across time points. The finding that mode of delivery independently predicted postpartum telomere length is noteworthy: caesarean birth is associated with greater inflammatory burden, prolonged recovery, and disrupted cortisol rhythms (Hyde et al 2012), any of which may accelerate postpartum telomere attrition. This observation warrants replication in larger cohorts and has implications for understanding differential maternal ageing trajectories by birth mode.

In addition to sleep deprivation, post-partum social interactions may also be important modulators of telomere dynamics. Negative social interactions in the first-year post-partum (including marital dissatisfaction and low perceived social support) are associated with greater telomere attrition, while positive interactions are protective (Houminer-Klepar et al. 2026). This finding underscores that the postpartum period involves a convergence of multiple psychosocial and physiological stressors that can shape maternal cellular ageing trajectory.

An increasingly important dimension of postpartum telomere biology is its relationship with perinatal mental health. Postpartum depression (PPD) has an estimated worldwide prevalence of 17.22% (Wang et al. 2021). A growing literature links psychiatric morbidity with accelerated cellular ageing (Talifu et al. 2025), and in turn PPD is associated with a greater risk of chronic diseases and multimorbidity in women’s mid-late life (Zhang et al. 2025). A 2024 cohort study using the UPPSAT (Uppsala-Personality and Sleep in the Postpartum Study) assessed TL at delivery in women with antenatal depression resolving postpartum, women with depression persisting to postpartum, and healthy controls. TL was negatively correlated with PPD symptom severity at pregnancy week 32 and at six weeks postpartum. Persistent PPD was associated with shorter TL, and adverse childhood experiences (ACE) moderated the association: among women with high ACE burden, persistent PPD predicted markedly shorter TL compared with controls. Importantly, neither *TERT* nor *TERC* genotype moderated the trajectory, suggesting that the observed TL differences reflect cumulative environmental rather than genetic influences (Vrettou et al. 2024).

Incollingo Rodriguez et al. (2022) demonstrated that acculturative stress in Latina mothers in the US, predicted shorter TL during pregnancy (assessed at 24–32 weeks gestation), particularly among women with high methylation of the *FOXP3* promoter region. Crucially, shorter pre-natal TL then predicted greater PPD symptom severity at four to six weeks postpartum, even after adjustment for baseline psychosocial variables. This finding, with shorter telomeres predicting later depression, positions cellular ageing not merely as a consequence of perinatal stress but as a potential upstream biomarker of vulnerability to PPD, with significant implications for early screening and intervention. Other anxiety and inflammatory markers are also associated with shorter LTL in the early postpartum period Groer et al. (2020). As such, any comprehensive model of postpartum telomere biology must account for social determinants of cellular ageing.

Collectively, these studies support a model in which the psychological and cellular ageing effects of perinatal stress are mutually reinforcing: psychological distress shortens telomeres, and shorter telomeres, perhaps through their association with impaired stress-system regulation and increased inflammation, predispose to further psychological deterioration. This bidirectional pathway may create a self-amplifying cycle during the postpartum period that has lasting consequences for maternal health.

### Peri-menopause, menopause and lifetime oestrogen exposure

Beyond pregnancy and the postpartum period, the peri-menopausal and menopausal transition represents a pivotal stage in which the cumulative effects of reproductive history and hormonal change can converge to shape cellular ageing. Shirazi et al. (2020) examined clinical composite measures of biological age rather than cellular measures, in a large US sample (n = 4418). Using four validated composite measures (the Levine Method, homeostatic dysregulation, the Klemera–Doubal method, and allostatic load), they found that parity exhibited a U-shaped relationship with biological age acceleration in post-menopausal but not pre-menopausal women, with ageing lowest among women reporting three to four live births. The absence of an effect in pre-menopausal women contrasts with the cellular findings described above. The authors propose that telomere length and epigenetic clocks may act as earlier, more sensitive indicators of reproductive costs, whereas clinical composite measures only capture these costs after menopause, when the putative compensatory mechanisms of the reproductive years are no longer operative. Telomere length and DNA methylation age are often poorly correlated with clinical measures of biological ageing, suggesting they index fundamentally different components of the ageing process.

Fan et al. (2023), drawing on a very large sample from the UK Biobank (n = 224,965), situated parity-related telomere shortening within a broader landscape of female reproductive factors. Several reproductive variables were significantly associated with shorter LTL after adjustment for confounders. Early menarche (before age 12) was linked to shorter telomeres, possibly through inflammation-related mechanisms. Early menopause (before age 45) and short reproductive lifespan (under 30 years) showed the strongest associations with shorter LTL, likely reflecting reduced lifetime oestrogen exposure, since oestrogen is thought to protect telomeres by stimulating telomerase activity. Multiparity and early age at first birth (before age 20) were both associated with shorter LTL, though the authors suggest these associations may be more strongly driven by socioeconomic and psychosocial factors than by reproductive biology directly. Use of both oral contraceptives and hormone replacement therapy were associated with shorter LTL, with longer duration and earlier initiation showing stronger effects. Notably, history of miscarriage and stillbirth were not significantly associated with LTL (Fan et al. 2023). The study identified nonlinear relationships between LTL and several reproductive timing variables, reinforcing the idea that the totality of a woman’s reproductive history, from menarche to menopause, shapes her cellular ageing trajectory.

Oestrogen is widely hypothesised to confer protection against cellular ageing through its stimulatory effect on telomerase. An oestrogen response element is present in the promoter of the catalytic subunit of telomerase (hTERT), and in vitro studies have demonstrated oestrogen-dependent upregulation of telomerase activity (Calado et al. 2009). Lin et al. (2011) examined this hypothesis in 53 postmenopausal women, finding that women with a longer reproductive lifespan (i.e. age between menarche and menopause) had a significantly longer LTL. This suggests that sustained endogenous oestrogen exposure is associated with more favourable telomere biology in post-menopause. Earlier work also demonstrated that women with the longest LTL underwent natural menopause approximately three years later than those with the shortest LTL, with each one-kilobase increase in LTL associated with an average 10.2-month delay in menopausal onset (Gray et al. 2014). This suggests a shared biological architecture linking telomere maintenance, ovarian reserve, and reproductive lifespan.

However, the Sister Study (Kresovich et al. 2018) reported apparently contradictory findings, with longer reproductive period (which entails greater cumulative oestrogen exposure) associated with shorter TL in postmenopausal women. Furthermore, large longitudinal data suggest that the rate of LTL attrition is actually faster during the pre-menopausal period than the post-menopausal period, casting doubt on direct oestrogenic telomerase stimulation as the primary explanation (Dalgard et al. 2015; Lapham et al. 2015). This suggests that the hormonal milieu of the fertile years is not unambiguously protective of telomeres. The overall physiological burden of reproductive activity (immune activation, oxidative stress, placentation-related cellular remodelling, and lactation-related energetic demands) may overwhelm any telomerase-stimulating benefit of oestrogen when considered across the full reproductive lifespan. Furthermore, no significant effect of exogenous oestrogen intake on LTL attrition rate has been demonstrated, suggesting the sex gap in TL originates earlier in life, possibly during foetal development, rather than emerging solely from premenopausal oestrogenic exposure (Lapham et al. 2015). Under this model, the lower TL often observed in postmenopausal parous women relative to nulliparous women of equivalent age may primarily reflect the accumulated costs of repeated pregnancy and lactation across the reproductive lifespan, rather than oestrogen withdrawal at menopause per se.

Premature menopause, defined as cessation of menses before age 40, has been independently associated with shorter LTL. A Mendelian randomisation study supports a causal contribution of telomere shortening to earlier onset of natural menopause, suggesting that accelerated telomere attrition may deplete ovarian follicular reserve, contributing to shortened reproductive lifespan (Schuermans et al. 2023). Women who undergo premature menopause have elevated risk of coronary artery disease, with LTL partially mediating the cardiovascular risk (Shadyab et al. 2017). This intersection of reproductive ageing, telomere biology, and cardiometabolic health represents an important frontier for understanding how the biological costs of reproductive history translate into long-term disease risk.

Overall, the evidence suggests that telomere length in later life reflects the integrated impact of reproductive timing, hormonal exposure, and the cumulative physiological costs of reproduction, positioning menopause as a key inflection point linking reproductive history to long-term cellular ageing and disease risk.

## Discussion

**Fig. 1:**
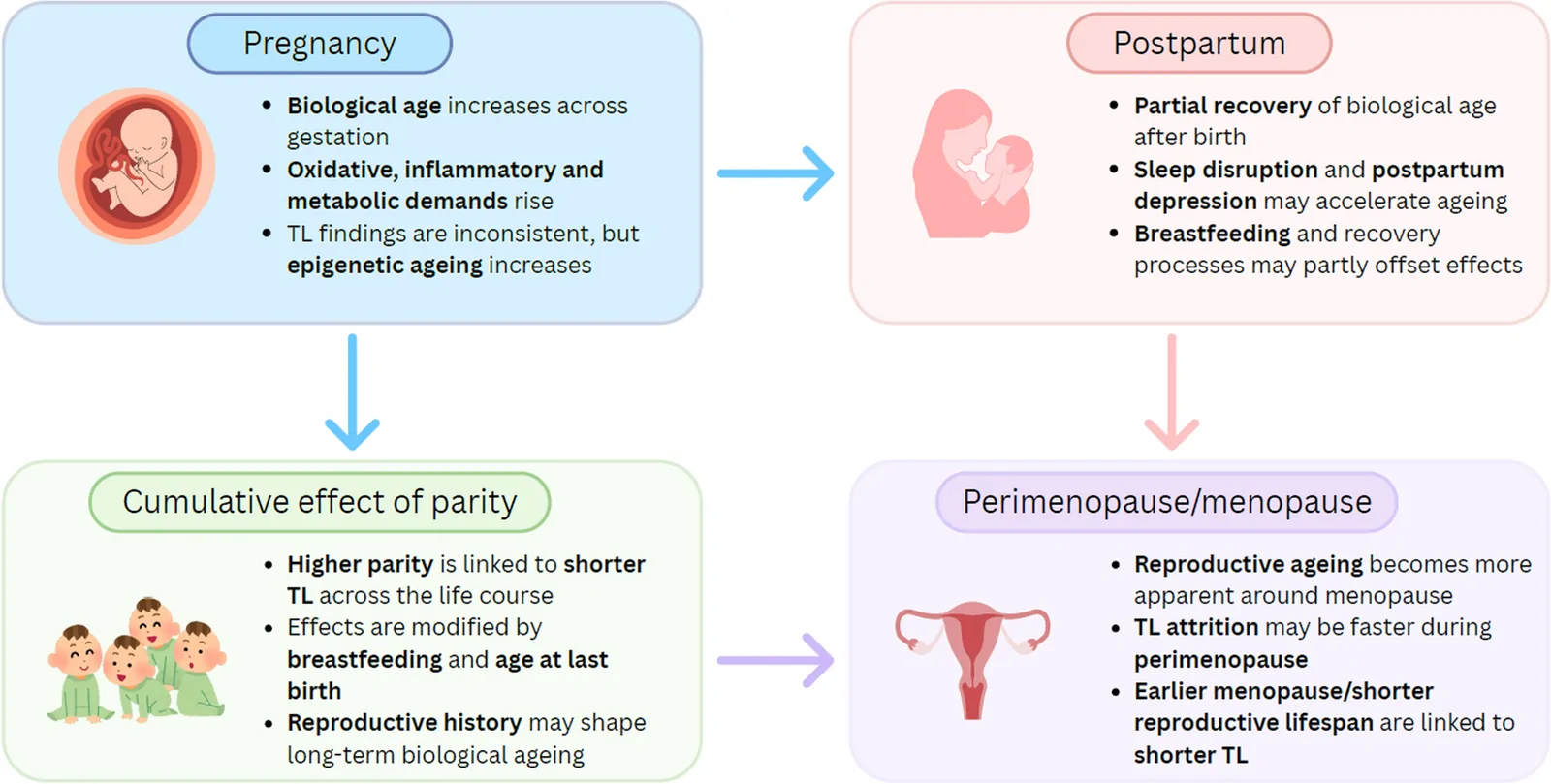
Reproductive events from pregnancy through to menopause are associated with cumulative changes in biological age, as indexed by telomere length and epigenetic clocks. Effects vary by reproductive stage and are modified by factors including breastfeeding, parity, and age at last birth. *TL* telomere length

The evidence reviewed here supports a biologically plausible and increasingly well-characterised link between pregnancy and postpartum stressors and accelerated telomere shortening and epigenetic ageing. The postpartum period is characterised by a convergence of factors known to influence telomere dynamics, including sleep disruption, hormonal fluctuations, psychological stress, immune activation, and the recovery demands of parturition (Bilodeau et al 2025). Together, these factors create a physiological environment that may promote accelerated cellular ageing during a critical window of maternal health (Fig. 1, Table 1). The framing of pregnancy itself as a transient model of accelerated biological ageing, with biological recovery that is partial and modifiable (Pham et al. 2024; Ryan et al. 2024), provides an important conceptual anchor for understanding why the postpartum period matters not just for immediate health but for the long-term ageing trajectory.

**Table 1:** Summary of studies examining associations between reproductive factors and telomere length (TL) or biological age in women

Cases		Controls		Population	Key finding	TL method	References
Type	N =	Type	N =				
Parous women (≥ 1 live birth)	1505	Nulliparous women	444	Nationally representative US women aged 20–44 (NHANES 1999–2002)	TL ↓ with parity	qPCR	Pollack et al. (2018)
N/A (cross sectional study)	N/A	N/A	N/A	Women aged 35–74 from the Sister Study (US), cancer-free at blood draw; predominantly White; n = 1,048	TL ↓ with higher parity, especially with 4–5 births TL ↓ with longer reproductive period TL ↑ with longer breastfeeding duration	qPCR	Kresovich et al. (2018)
N/A (cross sectional study)	N/A	N/A	N/A	Filipino women aged 20–22 from the Cebu Longitudinal Health and Nutrition Survey (CLHNS); n = 821 (TL), n = 397 (DNAmAge)	TL ↓ with gravidity DNAmAge ↑ with gravidity	qPCR	Ryan et al. (2018)
N/A (systematic review)	N/A	N/A	N/A	Women of reproductive age across multiple countries (14 studies; predominantly USA, also Canada, Central America, Philippines, Israel)	TL ↓ with parity (ns)	qPCR and Southern blot	Houminer–Klepar et al. (2023)
N/A – crosssectional and longitudinal cohort	N/A	N/A	N/A	Filipino adults (aged 20–22) from the CLHNS; 825 women and 910 men cross-sectionally; 331 women longitudinally	Epigenetic age ↑ with pregnancy number/gravidity	DNAmTL	Ryan et al. (2024)
N/A – prospective longitudinal cohort	N/A	N/A	N/A	119 low-risk pregnant women followed from early to late pregnancy and ~ 3 months postpartum (University of California Irvine cohort); US	Biological age ↑ across gestation, with reversal postpartum Recovery was linked to lower pre-pregnancy BMI and breastfeeding	Epigenetic clocks	Pham et al. (2024)
Women with later maternal age at last birth (≥ 30, ≥ 34, or ≥ 38 years)	300	Women whose last birth was by age 29	87	Elderly women (≥ 70 years) with familial longevity, non-Hispanic White, from the Long Life Family Study (LLFS)	TL ↑ in women who had their last child later in life (34–37 or over 38) vs those who had last child by 29	qPCR	Fagan et al. (2017)
Women with longer reproductive years (> 35 years; greater endogenous oestrogen exposure)	53 cases and controls	Women with shorter reproductive years (≤ 35 years)		Postmenopausal women (ages 49–69) at risk for cognitive decline, all on HT ≥ 1 year; predominantly Caucasian; US (UCSF/Stanford)	TL ↑ with longer reproductive lifespan	qPCR	Lin et al. (2011)
Women with natural menopause	486	Women with surgical menopause	179	White women aged ≥ 65 years from the Cardiovascular Health Study (CHS), a multi-site US longitudinal cohort	TL ↑ with later menopause	Southern blot	Gray et al. (2014)
N/A (longitudinal cohort study)	N/A	N/A	N/A	Danish twins (Danish National Twin Registry); 405 women (age 18–64) and 329 men (age 18–59) at baseline; ~ 12 year follow-up	TL attrition faster during premenopausal period than postmenopausal period; rate decelerated from pre- to peri- to post-menopause; sex difference in TL likely established prior to adulthood	Southern blot	Dalgard et al. (2015)
N/A (cohort study)	N/A	N/A		Postmenopausal women from a population-based cohort in Belgium	TL ↑ with later menopause	qPCR	Schuermans et al. (2023)
Individuals exposed to severe stress	85 in total	Recovery state/post stress comparison		Humans and/or mice across several stress and recovery models	Biological ageing ↑ with severe stress but can be partially restored	DNAm clocks	Poganik el al. (2023)
Pregnant women (cross-sectional study)	88	N/A		Racially diverse US women aged 18– 46, normative pregnancies, central North Carolina	TL ↑ with increased oxidative stress and IL-6	qPCR	Etzel et al. (2025)
Women with preeclampsia or spontaneous preterm birth	156	Women with uncomplicated term births	63	Postpartum women from diverse cohorts (suburban cohort n = 66, urban cohort n = 90)	TL differences ns	qPCR	Panelli et al (2024)
Postpartum women with short sleep (< 7 h/night) at 6 months	19	Postpartum women with sufficient sleep (≥ 7 h/night) at 6 months	14	Women aged 23–45, 1 year postpartum, racially diverse, western USA	TL ↓ with insufficient sleep	DNAm clocks	Carroll et al (2021)
Short sleep duration (≤ 6 h)	106,192	Normal sleep duration (7–8 h)	305,74 2	Mendelian randomisation study of individuals of European ancestry from UK Biobank	Genetically determined short sleep causally associated with shorter TL	qPCR	Hu et al. (2023)
Individuals with poor sleep	704	N/A	N/A	Adults from São Paulo, Brazil (EPISONO cohort), mean age 42.1 years at baseline, followed for 8 years	TL ↓ with poorer sleep	qPCR	Tempaku et al. (2025)
N.A (longitudinal cohort study of uncomplicated pregnancies)	N/A	N/A	N/A	46 women with singleton, term livebirths from US, enrolled 2012–2018; predominantly White (57.8%)	No clear gestational change in TL Postpartum TL ↓ after caesarean	qPCR	Panelli et al (2022)
N.A (longitudinal cohort study of uncomplicated pregnancies)	N/A	N/A	N/A	103 first-time mothers from Israel, mean age 28.6 years	TL ↑ with better social interactions/marital satisfaction	qPCR	Houminer–Klepar et al. (2026)
Women with PPD symptoms	96	Mothers without PPD	184	Pregnant/postpartum women from the BASIC cohort, Sweden	TL ↓ with PPD	qPCR	Vrettoi et al (2024)
N/A (longitudinal cohort stud)	N/A	N/A	N/A	150 healthy pregnant Latinx women living in North Carolina, USA	Shorter TL pre-natal predicted greater PPD symptoms Discrimination (acculturative stress) leads to TL attrition	qPCR	Incollingo et al. (2022)
N/A	N/A	N/A	N/A	197 postpartum women	TL ↓ with anxiety in post-partum mothers	qPCR	Groer et al. (2020)
N/A (crosssectional study)	N/A	N/A	N/A	1232 peri- and postmenopausal women aged	TL ↑ with later maternal age	qPCR	Latour et al. (2020)
N/A (crosssectional population study)	N/A	N/A	N/A	Women from the UK Biobank	TL ↓ with early menarche, early menopause, short reproductive lifespan, early age at first birth, multiparity, and use of oral contraceptives and hormone replacement therapy	qPCR	Fan et al. (2023)
N/A (cross sectional study)	N/A	N/A	N/A	2166 pre- and 2252 post-menopausal Women from NHANES 1999–2010	Agieng was *lowest* among women who had 3–4 children, and higher at both extremes (very few or many births)	Epigenetic clocks	Shirazi et al (2020)

Studies are grouped by reproductive domain: parity and gravidity; age at last birth and reproductive lifespan; menopausal status and timing; pregnancy and postpartum changes; and sleep, stress, and psychosocial factors in the perinatal period

TL direction arrows (↑/↓) indicate longer/shorter TL respectively, relative to the comparison group or per unit increase in the exposure

*NS* non-significant, *PPD* peri-partum depression

A central observation of this review is that the telomere biology of the postpartum period cannot be separated from the broader question of how reproductive history shapes biological ageing across the lifespan. Evidence from multiple large studies suggests that nulliparous women retain longer telomeres, and that this advantage is apparent as a population-level signal by mid-life and the menopausal transition (Pollacl et al. 2018; Panelli et al. 2022; Houminer–Klepar et al. 2023). The picture is far more nuanced, however. The timing of childbirth, total number of births, breastfeeding duration, mode of delivery, mental health history, socioeconomic context, and underlying genetic architecture all appear to modulate whether and how reproduction affects long-term TL. Women who give birth in their late thirties may actually have longer telomeres than those who gave birth younger, reflecting a selection effect of biological resilience (Fagan et al. 2017; Latour et al. 2020). Women who breastfeed for extended durations may partially offset the telomere costs of gestation (Pham et al. 2024). Women who undergo caesarean delivery may face an additional postpartum cellular ageing burden beyond that attributable to sleep loss alone (Panelli et al. 2022).

The weight of evidence from rigorously conducted studies suggests that higher parity is associated with shorter TL, particularly at four or more births and in older women (Pollack et al 2018; Kresovich et al. 2018; Ryan et al. 2024). The non-significant pooled effect in the 2023 meta-analysis by Houminer–Klepar et al. likely reflects genuine heterogeneity and the age-moderation of the effect rather than its absence; the age at which reproductive costs become biologically legible in TL may fall beyond the study windows of most existing cohorts.

The bidirectional relationship between postpartum mental health and telomere biology, demonstrated in Vrettou et al. (2024) and Incollingo Rodriguez et al. (2022), adds important complexity to the causal model. Cellular ageing does not merely reflect the downstream consequences of stress and sleep deprivation; shorter telomeres at baseline may constitute a vulnerability factor for PPD, creating a self-amplifying cycle of biological and psychological deterioration that, if unaddressed, could have lasting consequences for maternal wellbeing and, through intergenerational pathways, for offspring health. From a clinical perspective, these findings collectively make a strong case for elevating maternal health in postpartum system, which remain narrowly focused on physical wound healing and infant feeding (Ojomo et al. 2025). The addition of sleep and mental health screening, alongside meaningful social and structural support for new mothers, is supported not only by traditional psychological endpoints but now by biological ageing evidence.

The evidence reviewed here supports a graded, dose-dependent relationship between reproductive history and telomere shortening: nulliparous women appear to retain telomere length advantages into the menopausal transition, and each additional birth is associated, on average, with incrementally shorter telomeres in later life. These effects are modulated by a range of factors including timing of childbirth, breastfeeding duration, mode of delivery, mental health, and socioeconomic context, all of which must be considered in a comprehensive life-course framework.

### Methodological considerations

Interpreting the literature on TL and reproductive history requires careful attention to methodological heterogeneity. TL is most commonly measured in peripheral blood leukocytes by quantitative PCR (qPCR, reported as a T/S ratio) or by Southern blot analysis of terminal restriction fragments (TRFs) (Yu et al. 2024). These methods yield different absolute values and have different sensitivity profiles; qPCR is higher-throughput but subject to greater variability, while Southern blot provides absolute estimates at higher labour cost. More recently, DNAmTL offers the additional advantage of correlating TL with other epigenetic ageing markers in the same assay (Carroll et al. 2021).

Advances in epigenetic clock technology, particularly second-generation clocks (GrimAge, PhenoAge) and the third-generation DunedinPACE, have substantially expanded the toolkit available for measuring biological ageing in perinatal populations, and their application to postpartum cohorts has already yielded important insights. The consistent finding that postpartum sleep deprivation accelerates both epigenetic ageing and TL shortening, and that breastfeeding partially reverses pregnancy-associated biological ageing, positions these biomarkers not only as research endpoints but as potential targets for clinical intervention. The interpretation of epigenetic clock data in perinatal populations carries its own methodological challenges. Many clocks were trained on non-pregnant adult samples, and the physiological changes of pregnancy may influence DNA methylation independently of true biological ageing. Emerging work validating GrimAge and DunedinPACE in myometrial as well as peripheral blood tissue during pregnancy (Erickson et al. 2022) is an important step, but tissue-specific validation of epigenetic clock performance in postpartum populations remains an important research priority.

A further challenge is the definition of parity. Studies vary considerably in whether they count only live births, include stillbirths and miscarriages, or focus on gravidity. These distinctions matter because the physiological burden of pregnancy may contribute to telomere attrition regardless of outcome, and restricting parity counts to live births may underestimate the full reproductive cost. Additionally, most studies on parity and TL have been conducted in high-income countries with relatively low average parity; generalisability to populations where family sizes are larger, nutritional support lower, and reproductive burdens compounded by structural poverty remains uncertain.

Selection bias represents a further critical consideration. Women who remain childless may differ systematically from those who have children in ways that confound comparisons of TL, including socioeconomic status, health behaviours, chronic stress exposure, and access to healthcare. Studies that fail to adequately control for these covariates risk attributing to parity what is attributable to confounding factors. The strongest evidence comes from nationally representative samples with extensive covariate adjustment (e.g., Pollack et al. 2018) and from longitudinal designs with within-person TL measures (e.g., Ryan et al. 2018; Panelli et al. 2022). Future work should prioritise pre-conception TL assessment to enable within-person comparisons across reproductive events.

## Future directions

Future research should prioritise longitudinal studies that track telomere length and epigenetic clock measures from before conception through extended postpartum periods and beyond the menopausal transition, enabling a comprehensive understanding of how each reproductive event contributes cumulatively to biological ageing. Studies should include age- and socioeconomically matched nulliparous comparison groups to isolate the contribution of parity from confounding lifestyle and demographic factors; such comparisons are present in only a minority of existing studies. Pre-conception TL assessment should be included wherever possible to allow within-person longitudinal designs.

Mechanistic studies are needed to elucidate the specific pathways linking sleep disruption, mode of delivery, lactation, and parity with telomere maintenance and epigenetic age, particularly the relative contributions of inflammation, oxidative stress, hormonal dysregulation, and DNA damage response activation. The finding that caesarean delivery is independently associated with shorter postpartum LTL (Panelli et al. 2022) deserves replication and mechanistic follow-up, given rising global caesarean rates (Ojong et al. 2026).

Intervention studies aimed at improving postpartum sleep, whether through behavioural strategies, partner education, infant sleep interventions, or policy-level parental leave extensions, should include biological ageing biomarkers evidence alongside conventional psychological and cognitive outcomes. The potential for breastfeeding to partially reverse pregnancy-associated epigenetic ageing (Pham et al. 2024) suggests that feeding choices and their support infrastructure are relevant to maternal ageing trajectories and should be included in future intervention designs.

Greater attention to equity is essential. The biological ageing costs of reproduction are not uniformly distributed: they are compounded by racial discrimination, socioeconomic disadvantage, and structural barriers to healthcare access (Incollingo Rodriguez et al. 2022; Houminer–Kelper et al. 2023). Future studies should oversample marginalised populations, include detailed measures of allostatic load and social determinants of health, and explicitly examine whether interventions reduce health disparities in postpartum cellular ageing. Finally, the application of next-generation epigenetic clocks, particularly DunedinPACE, which quantifies the real-time pace of ageing rather than cumulative age, to postpartum cohorts with granular reproductive history data represents a high-priority methodological advance that could substantially clarify the dose-response relationships between reproductive events and maternal biological ageing.

Longitudinal, pre-conception-to-post-menopause studies of the kind proposed above would therefore not only refine postpartum maternal care, but would also generate a dataset capable of addressing several of biogerontology’s most persistent open questions concerning homeodynamic capacity, sex-specific longevity assurance, and the adaptive-versus-harmful classification of age-related molecular change.

## Data Availability

No datasets were generated or analysed during the current study.

## References

[CR1] Belsky D, Caspi A, Cocoran D, Sugden L, Poulton R, Arseneault L, Baccarelli A, Chamarti K, Gao X, Hannon E, Lee H, Harrington H, Houts R, Kothari M, Kwon D, Mill J, Scwartz J, Vokonas P, Wang C, Williams B, Moffitt T (2022) DunedinPACE, a DNA methylation biomarker of the pace of aging. Elife. 10.7554/eLife.73420 35029144 PMC8853656

[CR2] Bilodeau CC, Boudreau LE, Sharkey KM (2025) Breastfeeding and maternal sleep. In: Sharma V, Palagini L (eds) Sleep and perinatal psychiatric disorders. Springer

[CR3] Blackburn E, Epel E, Lin J (2015) Human telomere biology: a contributory and interactive factor in aging, disease risks, and protection. Science 350:1193–1198. 10.1126/science.aab3389 26785477

[CR4] Broderick M, Khan Q, Moradikor N (2025) Understanding the connection between stress and sleep: from underlying mechanisms to therapeutic solution. Prog Brain Res 291:137–159. 10.1016/bs.pbr.2025.01.016 40222777

[CR5] Calado RT, Young NS (2009) Telomere diseases. N Engl J Med 361(24):2353–2365. 10.1056/nejmra0903373 20007561 PMC3401586

[CR6] Carroll JE, Cole SW, Seeman TE, Breen EC, Witarama T, Arevalo JM, Irwin MR (2016) Partial sleep deprivation activates the DNA damage response (DDR) and the senescence-associated secretory phenotype (SASP) in aged adult humans. Brain Behav Immun 51:223–229 26336034 10.1016/j.bbi.2015.08.024 PMC4679552

[CR7] Carroll JE, Ross KM, Horvath S, Okun M, Hobel C, Rentscher KE, Coussons-Read M, Dunkel Schetter C (2021) Postpartum sleep loss and accelerated epigenetic aging. Sleep Health 7(3):362–367. 10.1016/j.sleh.2021.02.002 33903077 PMC10027398

[CR8] Cawthon RM, Smith KR, O’Brien E, Sivatchenko A, Kerber RA (2003) Association between telomere length in blood and mortality in people aged 60 years or older. Lancet 61(9355):393–395. 10.1016/s0140-6736(03)12384-7 12573379

[CR9] Dalgard C, Benetos A, Verhulst S, Labat C, Kark JD, Christensen K, Kimura M, Kyvik KO, Aviv A (2015) Leukocyte telomere length dynamics in women and men: menopause vs age effects. Int J Epidemiol 44(5):1688–1695. 10.1093/ije/dyv165 26385867 PMC4681111

[CR10] Epel ES, Blackburn EH, Lin J, Dhabhar FS, Adler NE, Morrow JD, Cawthon RM (2004) Accelerated telomere shortening in response to life stress. Proc Natl Acad Sci U S A 101(49):17312–17315. 10.1073/pnas.0407162101 15574496 PMC534658

[CR11] Erickson E, Knight A, Smith A, Myatt L (2022) Advancing understanding of maternal age: correlating epigenetic clocks in blood and myometrium. Epigenet Commun. 10.1186/s43682-022-00010-0 PMC9432845 36052275

[CR12] Etzel L, Ye Q, Apsley AT, Chiaro C, Petri LE, Kozlosky J, Propper C, Mills-Koonce R, Short SJ, Garrett-Peters P, Shalev I (2025) Maternal telomere length and oxidative stress in pregnancy: cross-sectional analysis with an exploratory examination of systemic inflammation. BMC Pregnancy Childbirth 25(1):395. 10.1186/s12884-025-07542-y 40186152 PMC11971816

[CR13] Fagan R, Sun F, Bee H, Elo I, Andersen S, Lee J, Christensen K, Thyagarajanm B, Sebastiani P, Perls T, Honig L, Schupf N (2017) Telomere length Is longer in women with late maternal age. Menopause 24(5):497–501. 10.1097/GME.0000000000000795 27922939 PMC5403597

[CR14] Fan G, Liu Q, Bi J, Qin X, Fang Q, Wang Y, Song L (2023) Association between female-specific reproductive factors and leukocyte telomere length. Hum Reprod 38(11):2239–2246. 10.1093/humrep/dead176 37671590

[CR15] Ferrari L, Comotti A, Fattori A, Barnini T, Laurino M, Bufano P, Albetti B, Hoxha M, Russo S, Ciocan C, Bonzini M (2025) Impact of night shift work on telomere length and epigenetic age in older workers. J Occup Med Toxicol. 10.1186/s12995-025-00477-2 41035087 PMC12487556

[CR16] Fisher JJ, Bartho LA, Perkins AV, Holland OJ (2020) Placental mitochondria and reactive oxygen species in the physiology and pathophysiology of pregnancy. Clin Exp Pharmacol Physiol 47:176–184. 10.1111/1440-1681.13172 31469913

[CR17] Gay CL, Lee KA, Lee SY (2004) Sleep patterns and fatigue in new mothers and fathers. Biol Res Nurs 5(4):311–318. 10.1177/1099800403262142 15068660 PMC1307172

[CR18] Gray K, Schiff M, Fitzpatrick A, Kimura M, Aviv A, Starr J (2014) Leukocyte telomere length and age at menopause. Epidemiology 25(1):139–146. 10.1097/ede.0000000000000017 24213145 PMC3926311

[CR19] Groer M, Louis-Jacques A, Szalacha L, Redwine L, Dracxler R, Keefe D (2020) Relationship of anxiety, inflammation, and telomere length in postpartum women: a pilot study. Biol Res Nurs 22(2):256–262. 10.1177/1099800419890424 31858822 PMC7273803

[CR20] Gruber H-J, Semeraro MD, Renner W, Herrmann M (2021) Telomeres and age-related diseases. Biomedicines 9(10):1335–1354. 10.3390/biomedicines9101335 34680452 PMC8533433

[CR21] Grzeszczak K, Łanocha-Arendarczyk N, Malinowski W, Ziętek P, Kosik-Bogacka D (2023) Oxidative stress in pregnancy. Biomolecules 13(12):1768–1791. 10.3390/biom13121768 38136639 PMC10741771

[CR22] Houminer-Klepar N, Bord S, Epel E, Baron-Epel O (2023) Are pregnancy and parity associated with telomere length? A systematic review. BMC Pregnancy Childbirth 23(1):733. 10.1186/s12884-023-06011-8 37848852 PMC10583451

[CR23] Houminer-Klepar N, Bord S, Epel E, Lin J, Sagi S, Baron-Epel O (2026) Social interactions and cell aging dynamics in postpartum women. Biol Res Nurs 28(2):205–217. 10.1177/10998004251397544 41230634 PMC12929666

[CR24] Hu J, Lu J, Lu Q, Weng W, Guan Z, Wang Z (2023) Mendelian randomization and colocalization analyses reveal an association between short sleep duration or morning chronotype and altered leukocyte telomere length. Commun Biol 6:1014. 10.1038/s42003-023-05397-7 37803147 PMC10558505

[CR25] Hyde MJ, Mostyn A, Modi N, Kemp PR (2012) The health implications of birth by Caesarean section. Biol Rev 87:229–243. 10.1111/j.1469-185X.2011.00195.x 21815988

[CR26] Incollingo Rodriguez AC, Polcari JJ, Nephew BC, Harris R, Zhang C, Murgatroyd C, Santos HP (2022) Acculturative stress, telomere length, and postpartum depression in Latinx mothers. J Psychiatr Res 147:301–306. 10.1016/j.jpsychires.2022.01.063 35123339 PMC8882151

[CR27] Keefe D, Marquard K, Liu L (2006) The telomere theory of reproductive senescence in women. Curr Opin Obstet Gynecol 18(3):280–285. 10.1097/01.gco.0000193019.05686.49 16735827

[CR28] Kirkwood TBL, Rose MR (1991) Evolution of senescence: late survival sacrificed for reproduction. Philos Trans R Soc B Biol Sci 332(1262):15–24. 10.1098/rstb.1991.0028 1677205

[CR29] Knight O, Hipp H, Abhari S, Gerkowicz S, Katler Q, McKenzie L, Shang W, Smith A, Spencer J (2022) Markers of ovarian reserve are associated with reproductive age acceleration in granulosa cells from IVF patients. Hum Reprod 37(10):2338–2345. 10.1093/humrep/deac178 PMC9527469 35944168

[CR30] Kresovich JK, Parks CG, Sandler DP, Taylor JA (2018) Reproductive history and blood cell telomere length. Aging (Albany NY) 10(9):2383–2393. 10.18632/aging.101558 30243019 PMC6188490

[CR31] Lapham K, Kvale MN, Lin J, Connell S, Croen LA, Dispensa BP, Risch N (2015) Leukocyte telomere length dynamics in women and men: menopause vs age effects. Int J Epidemiol 44(5):1688–1695. 10.1093/ije/dyv165 26385867 PMC4681111

[CR32] Latour C, O’Connell BA, Kelli M, Romano M, Kantor E, Mengmeng S (2020) Maternal age at last birth and leukocyte telomere length in a nationally representative population of perimenopausal and postmenopausal women. Menopause 27(11):1242–1250. 10.1097/gme.0000000000001669 33110040 PMC8448037

[CR33] Levine ME, Lu AT, Quach A, Chen BH, Assimes TL, Bandinelli S, Hou L, Baccarelli AA, Stewart JD, Li Y, Whitsel EA, Wilson JG, Reiner AP, Aviv A, Lohman K, Liu Y, Ferrucci L, Horvath S (2018) An epigenetic biomarker of aging for lifespan and healthspan. Aging (Albany NY) 10(4):573–591. 10.18632/aging.101414 29676998 PMC5940111

[CR34] Li A, Koch Z, Ideker T (2022) Epigenetic aging: biological age prediction and informing a mechanistic theory of aging. J Intern Med 292:733–744. 10.1111/joim.13533 35726002

[CR35] Lin J, Kroenke CH, Epel E, Kenna HA, Wolkowitz OM, Blackburn E, Rasgon NL (2011) Greater endogenous estrogen exposure is associated with longer telomeres in postmenopausal women at risk for cognitive decline. Brain Res 1379:224–231. 10.1016/j.brainres.2010.10.033 20965155 PMC3057451

[CR36] Lu AT, Quach A, Wilson JG, Reiner AP, Aviv A, Raj K, Hou L, Baccarelli AA, Li Y, Stewart JD, Whitsel EA, Assimes TL, Ferrucci L, Horvath S (2019a) DNA methylation GrimAge strongly predicts lifespan and healthspan. Aging (Albany NY) 11(2):303–327. 10.18632/aging.101684 30669119 PMC6366976

[CR37] Lu AT, Seeboth A, Tsai PC, Sun D, Quach A, Reiner AP, Kooperberg C, Ferrucci L, Hou L, Baccarelli AA, Li Y, Harris SE, Corley J, Taylor A, Deary IJ, Stewart JD, Whitsel EA, Assimes TL, Chen W, Li S, Horvath S (2019b) DNA methylation-based estimator of telomere length. Aging (Albany NY) 11(16):5895–5923. 10.18632/aging.102173 31422385 PMC6738410

[CR38] Ojomo O, Atibioke O, Alesinloye-King O, Erlandsson K, Ängeby K, Envall N (2025) A scoping study of postpartum mental health problems and associated factors: opportunities for research and practice. Discover Mental Health 5(1):136. 10.1007/s44192-025-00278-3 40921936 PMC12417353

[CR39] Ojong S, Temmerman M, Nsahlai C, Gidion D, Kihara A (2026) Why do cesarean delivery rates persistently rise despite evidence-based efforts to reduce them? Am J Obstet Gynecol 233(6):S569–S580. 10.1016/j.ajog.2025.08.014 41485842

[CR40] Panelli DM, Wang X, Mayo J, Wong RJ, Hong X, Becker M, Bianco K (2024) Association of pregnancy complications and postpartum maternal leukocyte telomeres in two diverse cohorts: a nested case-control study. BMC Pregnancy Childbirth 24(1):490. 10.1186/s12884-024-06688-5 39033276 PMC11264806

[CR41] Panelli DM, Leonard SA, Wong RJ, Becker M, Feyaerts D, Marić I, Bianco K (2022) Leukocyte telomere dynamics across gestation in uncomplicated pregnancies and associations with stress. BMC Pregnancy Childbirth 10.1186/s12884-022-04693-0 PMC9063069 35501726

[CR42] Pham H, Thompson-Felix T, Czamara D, Rasmussen JM, Lombroso A, Entringer S, Binder EB, Wadhwa PD, Buss C, O’Donnell KJ (2024) The effects of pregnancy, its progression, and its cessation on human (maternal) biological aging. Cell Metab 36(5):877–878. 10.1016/j.cmet.2024.02.016 38521058 PMC11774303

[CR43] Phillippe M (2022) Telomeres, oxidative stress, and timing for spontaneous term and preterm labor. Am J Obstet Gynecol 227(2):148–162. 10.1016/j.ajog.2022.04.024 35460626

[CR44] Poganik JR, Zhang B, Baht GS, Tyshkovskiy A, Deik A, Kerepesi C, Yim SH, Lu AT, Haghani A, Gong T, Hedman AM, Andolf E, Pershagen G, Almqvist C, Clish CB, Horvath S, White JP, Gladyshev VN (2023) Biological age is increased by stress and restored upon recovery. Cell Metab 35(5):807–820. 10.1016/j.cmet.2023.03.015 37086720 PMC11055493

[CR45] Pollack AZ, Rivers K, Ahrens KA (2018) Parity associated with telomere length among US reproductive age women. Hum Reprod 33(4):736–744. 10.1093/humrep/dey024 29452389

[CR46] Prather AA, Puterman E, Lin J, O’Donovan A, Krauss J, Tomiyama AJ, Epel ES, Blackburn EH (2011) Shorter leukocyte telomere length in midlife women with poor sleep quality. J Aging Res 2011:721390. 10.4061/2011/721390 22046530 PMC3199186

[CR47] Rattan SIS (2024) Seven knowledge gaps in modern biogerontology. Biogerontology 25:1–8. 10.1007/s10522-023-10089-0 38206540

[CR48] Ross K, Carroll J, Hovarth S, Hobel C, Coussens-Read M, Schetter C (2024) Epigenetic age and pregnancy outcomes: GrimAge acceleration is associated with shorter gestational length and lower birthweight. Clin Epigenetics 12:120. 10.1186/s13148-020-00909-2 PMC7409637 32762768

[CR49] Ryan C, Hayes G, Lee N, McDade T, Jones M, Kobor M, Kuzawa C, Eisenberg D (2018) Reproduction predicts shorter telomeres and epigenetic age acceleration among young adult women. Sci Rep 8(1):11100. 10.1038/s41598-018-29486-4 30038336 PMC6056536

[CR50] Ryan CP, Lee NR, Carba DB, MacIsaac JL, Lin DTS, Atashzay P, Belsky DW, Kobor MS, Kuzawa CW (2024) Pregnancy is linked to faster epigenetic aging in young women. Proc Natl Acad Sci U S A 121(16):e2317290121. 10.1073/pnas.2317290121 38588424 PMC11032455

[CR51] Sabot D, Lovegrove R, Stapleton P (2024) The association between sleep quality and telomere length: A systematic review. Brain, Behavior, and Immunity—Health 27 100566. 10.1016/j.bbih.2022.100577 PMC9860369 36691437

[CR52] Schuermans A, Nakao T, Uddin M, Hornsby W, Ganesh S, Shadyab A, Haring B, Shufelt C, Taub M, Mathias R, Kooperberg C, Reiner A, Bick A, Manson J, Natarajan P, Honigber M (2023) Age at menopause, leukocyte telomere length, and coronary artery disease in postmenopausal women. Circulat Res. 10.1161/CIRCRESAHA.123.322984 37489536 PMC10528840

[CR53] Shadyab AH, Macera CA, Shaffer RA, Jain S, Gallo LC, Waring ME, LaCroix AZ (2017) Ages at menarche and menopause and reproductive lifespan as predictors of exceptional longevity in women: the Women’s Health Initiative. Menopause 24(1):35–44. 10.1097/gme.0000000000000710 27465713 PMC5177476

[CR54] Shirazi TN, Hastings WJ, Rosinger AY, Ryan CP (2020) Parity predicts biological age acceleration in post-menopausal, but not pre-menopausal, women. Sci Rep 10(1):20522. 10.1038/s41598-020-77082-2 33239686 PMC7689483

[CR55] Talay A, Belikov AV, To PKP et al (2026) Open problems in ageing science: a roadmap for biogerontology. GeroScience 48:3351–3360 41205028 10.1007/s11357-025-01964-4 PMC13356218

[CR56] Talifu Z, Ren Z, Chen C, Guo S, Wu Y, Li Y, Su B, Zheng X (2025) The association between accelerated biological aging and the physical, psychological, and cognitive multimorbidity and life expectancy: cohort study. Aging Cell 24:e70142. 10.1111/acel.70142 40652396 PMC12419857

[CR57] Tempaku PF, D’Almeida V, Anderson M, Tufik S (2025) Sleep and telomere attrition: A longitudinal population-based study. J Sleep Res 34(1):e14274. 10.1111/jsr.14274 39054789

[CR58] Thomas N, Hudaib A-R, Romano-Silva M, Bozaoglu K, Thomas E, Rossel S, Kulkani J, Gurvich C (2021) Influence of cortisol awakening response on telomere length: Trends for males and females. Eur J Neurosci 55(9):2794–2803. 10.1111/ejn.14996 33012014

[CR59] Von Zglinicki T (2002) Oxidative stress shortens telomeres. Trends Biochem Sci 27(7):339–344. 10.1016/s0968-0004(02)02110-2 12114022

[CR60] Vrettoi M, Lager S, Toffoletto S, Iliadis S, Kallak T, Agnafors S, Nieratschker VM, Skalkidou A, Comasco E (2024) Peripartum depression symptom trajectories, telomere length and genotype, and adverse childhood experiences. BMC Psych. 10.1186/s12888-024-06115-1 PMC11462957 39379870

[CR61] Wang ZY, Liu JY, Shuai H, Cai ZX, Fu X, Liu Y, Xiao X, Zhang W, Krabbendam E, Liu S, Liu Z, Li Z, Yang B (2021) Mapping global prevalence of depression among postpartum women. Transl Psychiatry 11(1):543. 10.1038/s41398-021-01663-6 34671011 PMC8528847

[CR62] Wang J, Han T, Zhu X (2024) Role of maternal–fetal immune tolerance in the establishment and maintenance of pregnancy. Chin Med J 137(12):1339–1406. 10.1097/cm9.0000000000003114 PMC11188918 38724467

[CR63] Ye Q, Apsley AT, Etzel L, Hastings WJ, Kozlosky JT, Walker C, Wolf SE, Shalev I (2023) Telomere length and chronological age across the human lifespan: a systematic review and meta-analysis of 414 study samples including 743,019 individuals. Ageing Res Rev 90:102031. 10.1016/j.arr.2023.102031 37567392 PMC10529491

[CR64] Yu H, Byun Y, Park C (2024) Techniques for assessing telomere length: a methodological review. Comput Struct Biotechnol J 23:1489–1498. 10.1016/j.csbj.2024.04.011 38633384 PMC11021795

[CR65] Zhang J, Rane G, Dai X, Shanmugam M, Arfuso F, Perumal R, Lai M, Kappei D, Prem A, Sethi K (2016) Ageing and the telomere connection: An intimate relationship with inflammation. Ageing Res Rev 25:55–69. 10.1016/j.arr.2015.11.006 26616852

[CR66] Zhang Y, Cheng Y, Carrillo-Larco RM et al (2025) Postpartum depression in relation to chronic diseases and multimorbidity in women’s mid-late life: A prospective cohort study of UK Biobank. BMC Med 23:24. 10.1186/s12916-025-038 39838355 PMC11752811

